# Multiple Mechanisms Contribute to Centriole Separation in *C. elegans*

**DOI:** 10.1016/j.cub.2013.06.043

**Published:** 2013-07-22

**Authors:** Gabriela Cabral, Sabina Sanegre Sans, Carrie R. Cowan, Alexander Dammermann

**Affiliations:** 1 Max F. Perutz Laboratories, University of Vienna, Doktor-Bohr-Gasse 9, 1030 Vienna, Austria; 2 Research Institute of Molecular Pathology, Doktor-Bohr-Gasse 7, 1030 Vienna, Austria

## Abstract

Centrosome function in cell division requires their duplication, once, and only once, per cell cycle. Underlying centrosome duplication are alternating cycles of centriole assembly and separation [1]. Work in vertebrates has implicated the cysteine protease separase in anaphase-coupled centriole separation (or disengagement) and identified this as a key step in licensing another round of assembly [2]. Current models have separase cleaving a physical link between centrioles, potentially cohesin [3, 4], that prevents reinitiation of centriole assembly unless disengaged. Here, we examine separase function in the *C. elegans* early embryo. We find that depletion impairs separation and consequently duplication of sperm-derived centrioles at the meiosis-mitosis transition. However, subsequent cycles proceed normally. Whereas mitotic centrioles separate in the context of cortical forces acting on a disassembling pericentriolar material, sperm centrioles are not associated with significant pericentriolar material or subject to strong forces. Increasing centrosomal microtubule nucleation restores sperm centriole separation and duplication in separase-depleted embryos, while forced pericentriolar material disassembly drives premature separation in mitosis. These results emphasize the critical role of cytoskeletal forces and the pericentriolar material in centriole separation. Separase contributes to separation where forces are limited, offering a potential explanation for results obtained in different experimental models [5–7].

## Highlights

• Proximity, not linkage, controls centriole duplication licensing in *C. elegans*
• Separase is largely dispensable for centriole separation in the early embryo
• Key role for pericentriolar material in centriole cohesion

## Results and Discussion

Our understanding of the molecular mechanisms underlying centriole assembly has grown rapidly in recent years, with the *C. elegans* early embryo proving to be a fruitful model to study this process [8]. The core machinery for centriole assembly appears to be largely conserved across evolution. Whether this is also true for the regulatory steps limiting centriole assembly to once per cell cycle is not known. In vertebrates, initiation of centriole assembly has been shown to involve separase-mediated disengagement of the previous mother-daughter centriole pair [2], an event that normally occurs coincident with sister chromatid separation in anaphase.

In order to examine separase function in *C. elegans*, we raised antibodies against the *C. elegans* ortholog SEP-1. Consistent with previous reports [9, 10], we observed separase at multiple structures in the early embryo (Figure S1A available online): during meiosis, separase localized to meiotic chromosomes, the surrounding spindle, as and the cell cortex. Interestingly, separase also localized to the sperm pronucleus while being excluded from interphase nuclei at later stages. We hypothesize this to be due to the lack of a nuclear envelope around sperm chromatin immediately after fertilization [11]. It is difficult to discern any localization of separase to centrioles during meiosis. However, at later stages, separase localized weakly to the pericentriolar material throughout the cell cycle. Finally, separase was found concentrated on the centromeres of the holocentric chromosomes during prometaphase/metaphase before spreading throughout the chromatin in anaphase. To our knowledge, this is the first report of a shift in localization at the metaphase-anaphase transition, which may reflect separase function in sister chromatid resolution. This shift could also be seen in a GFP-tagged strain previously used to examine separase localization [9] (Figure S1C). Importantly, RNA interference (RNAi)-mediated depletion confirmed the specificity of our antibody signal, as well as adequate depletion of the target protein (Figure S1B).

**Figure 1:**
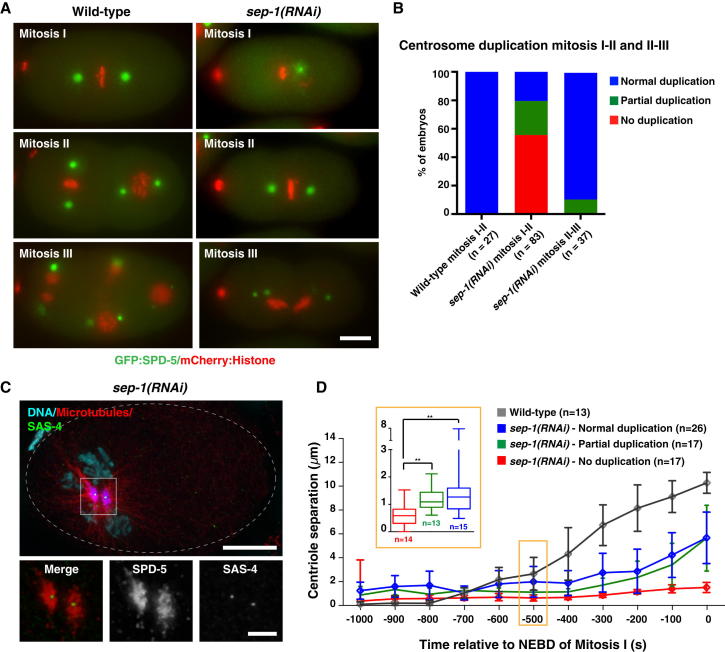
Characterization of the *sep-1(RNAi)* Phenotype (A) Stills from time-lapse movies of wild-type and *sep-1(RNAi)* embryos expressing GFP:SPD-5 and mCherry:Histone, acquired on a wide-field microscope. (B) Quantitation of centrosome duplication phenotype from time-lapse sequences as in (A). Centrosome duplication was scored as “partial” if some but not all centrosomes duplicated from one cell cycle to the next (e.g., an increase from two to three centrosomes between mitosis I and II, rather than the expected four). Note that a doubling of centrosome numbers from mitosis II to mitosis III would be considered “normal duplication” even if the starting number was abnormal (i.e., two or three). (C) Immunofluorescence micrograph of *sep-1(RNAi)* embryo stained for DNA, microtubules, pericentriolar material (SPD-5), and centrioles (SAS-4). (D) Centriole-to-centriole distance during the first mitotic division in control and *sep-1(RNAi)* embryos. Distances were measured from time-lapse sequences as in (A), with *sep-1(RNAi)* embryos divided into three classes according to duplication outcome in mitosis II. Error bars represent the 95% confidence interval. The box plot shows range, as well as upper and lower quartiles, for distances in mid-S phase (500 s before nuclear envelope breakdown [NEBD]). Asterisks indicate statistically significant differences between the three categories (Student’s t test, p < 0.05). The apparent premature separation in *sep-1(RNAi)* embryos prior to 700 s reflects cell cycle delays linked to separase depletion (see Figures S4A and S4B). Scale bars in (A) and (C) represent 10 μm, or 2.5 μm in the insets in (C). See also Table S1.

Previous studies of separase in *C. elegans* reported defects in meiotic and mitotic chromosome segregation, cortical granule exocytosis during eggshell formation, and cytokinesis after depletion or mutation of SEP-1 [9, 10, 12]. More recently, separase and meiosis-specific cohesin have also been linked to maintenance of centriole cohesion during male meiosis [13]. Here we set out to examine separase function in centrosome duplication in the early embryo. For this, we constructed a strain coexpressing GFP:SPD-5 and mCherry:H2B to monitor centrosomes and chromosomes and filmed embryos under conditions that support development of embryos with compromised eggshells. In wild-type embryos, sperm-derived centrioles separate shortly after completion of meiosis II. New daughter centrioles assemble alongside each parental centriole in a process that is complete by metaphase of the first mitosis. Mother and daughter centrioles separate during anaphase and the cycle repeats [14]. In *sep-1(RNAi)* embryos, separation of sperm-derived centrioles frequently failed, resulting in formation of monopolar spindles (n = 100/126 embryos) containing two discrete centrosomes (Figures 1A and 1C). A fraction of spindles (52/100) eventually bipolarized, and embryos with bipolar spindles attempted chromosome segregation in anaphase without separating sister chromatids (Figure 1A and data not shown). In all embryos, pericentriolar material disassembly occurred normally upon mitotic exit, and centrosomes moved apart. As previously reported, cytokinesis frequently failed such that successive mitoses occurred in a single cell. Were the separase phenotype limited to cytokinesis failure alone, we would expect to see a doubling of centrosome numbers in each mitosis: two in mitosis I, four in mitosis II, and eight in mitosis III. Strikingly, many embryos contained only two centrosomes in mitosis II, although these were always fully separated. Embryos with three or four centrosomes were also observed, indicating partial and occasionally complete duplication. In subsequent divisions, centrosome separation and duplication proceeded normally (Figures 1A and 1B). Late-stage embryos therefore accumulated multiple centrosomes, consistent with previous reports [12]. Previous analyses had identified two homologs of separase in *C. elegans*, *sep-1* and a nonessential gene, *zk430.5* [12]. Codepletion of *zk430.5* with *sep-1* did not enhance any aspect of the *sep-1* phenotype, ruling out redundant functions (Figure S2A). A second double-stranded RNA targeting a nonoverlapping part of the *sep-1* messenger RNA further confirmed specificity of the RNAi phenotype (Figure S2A).

**Figure 2:**
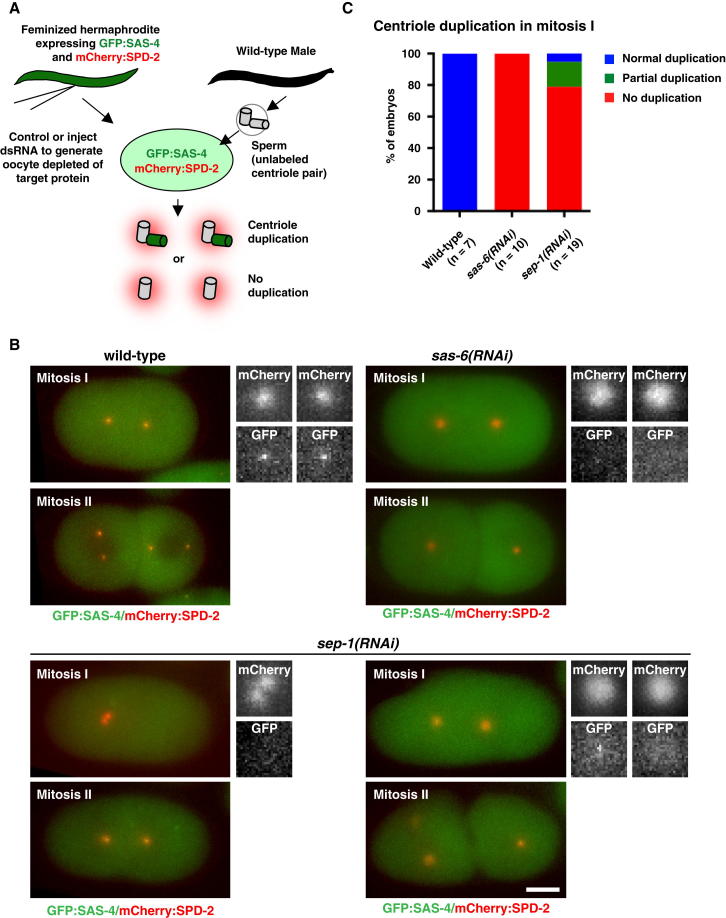
Centriole Duplication Fails in *sep-1(RNAi)* Embryos (A) Schematic of live assay for centriole duplication, based on incorporation of SAS-4 into newly assembling centrioles. (B) Results of experiments performed as outlined in (A) on control embryos, as well as embryos depleted of SAS-6 (control) and SEP-1 by RNAi. Stills are from wide-field time-lapse sequences, showing GFP:SAS-4 and mCherry:SPD-2 recruitment in mitosis I and II. *sep-1(RNAi)* panels illustrate failed (left) and partial duplication (right). (C) Quantitation of centriole duplication assay performed in (B). Scale bars in (B) represent 10 μm; insets are magnified 4×.

These results point to a defect in centriole assembly that is limited to the first cell cycle. To monitor centriole assembly in living embryos and correlate it with centrosome duplication, we constructed a strain coexpressing GFP:SAS-4 and mCherry:SPD-2 in a temperature-sensitive female-sterile background. Mating of feminized hermaphrodites with wild-type males introduces unlabeled sperm centrioles into an oocyte cytoplasm containing both fluorescent fusions (Figure 2A). SAS-4 stably incorporates during centriole assembly and does not exchange with the cytoplasmic pool. Recruitment therefore indicates assembly of new daughter centrioles [15, 16]. By contrast, SPD-2 localizes to both centrioles and pericentriolar material and rapidly exchanges with the cytoplasmic pool ([17] and our unpublished data). SPD-2 therefore labels all centrioles, whether sperm derived or assembled in the embryo cytoplasm. Using this assay, we found that GFP:SAS-4 recruitment and thus centriole assembly frequently failed in *sep-1(RNAi)* embryos (Figures 2B and 2C). Following embryos into the second division, we found the number of GFP:SAS-4 foci to be an accurate predictor of the number of additional centrosomes in the second mitosis. Thus, failure of centrosome duplication in separase-depleted embryos results from a failure of centriole assembly specifically in the first cell cycle (see also Figure S2B). Further experiments showed that failure occurs at the initiation step, with recruitment of the central tube/cartwheel component SAS-6 impaired in *sep-1(RNAi)* embryos (Figures S2C–S2E).

At first glance, these results appear to be similar to those obtained in *Xenopus* extracts [2]. Upon closer inspection, several differences become apparent. First, centrioles do disengage after *sep-1(RNAi)*. Whereas engaged centrioles (such as the mother-daughter centriole pair in metaphase embryos) appear as a single diffraction-limited spot by conventional light microscopy, sperm-derived centrioles in *sep-1(RNAi)* embryos are several microns apart, each surrounded by its own noncontiguous pericentriolar material (Figure 1C). A connecting linker would have to be highly flexible to allow this degree of separation. Second, the likelihood of centriole duplication correlated with distance between parental centrioles during S phase (Figure 1D). Such a proximity dependence is difficult to reconcile with a physical block to reduplication. Third, separase function appears to be critical for centriole separation only in the transition from the meiotic to mitotic cell cycle, while subsequent centrosome duplication cycles occurred normally, despite continued failure of chromosome segregation. This suggests the existence of additional mechanisms controlling centriole separation.

**Figure 3:**
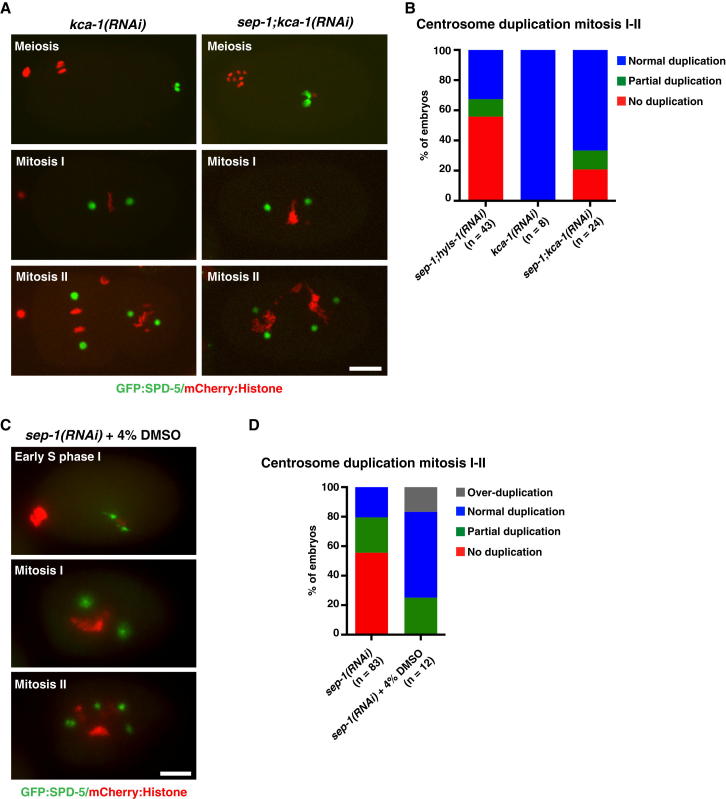
Rescue of *sep-1(RNAi)* Centrosome Duplication Phenotype by Increased Microtubule-Dependent Forces (A) Stills from spinning-disk confocal time-lapse movies of *kca-1(RNAi)* and *sep-1;kca-1(RNAi)* embryos expressing GFP:SPD-5 and mCherry:Histone. (B) Quantitation of centrosome duplication phenotype in *kca-1(RNAi)* and *sep-1;kca-1(RNAi)* embryos. *sep-1;hyls-1(RNAi)* data are shown for comparison. Differences in duplication outcome between *sep-1;kca-1(RNAi)* and *sep-1;hyls-1(RNAi)* are statistically significant (Chi-square test, p < 0.05). (C) Stills from a wide-field time-lapse movie of *sep-1(RNAi)* embryo expressing GFP:SPD-5 and mCherry:Histone treated with 4% dimethyl sulfoxide (DMSO). (D) Quantitation of centrosome duplication phenotype in DMSO-treated *sep-1(RNAi)* embryos. Untreated *sep-1(RNAi)* embryo data are shown for comparison. Differences in duplication outcome are statistically significant compared to *sep-1(RNAi)* alone (Chi-square test, p < 0.05). Scale bars in (A) and (C) represent 10μm.

In anaphase of mitosis, centrioles separate concomitant with disassembly of the pericentriolar material, which is being torn apart by pulling forces exerted through microtubule-cortex interactions [18]. At the meiosis-mitosis transition, sperm-derived centrioles are associated with little pericentriolar material and few microtubules and are thus unlikely to experience strong pulling forces. Depletion of what little pericentriolar material is present at this stage with *spd-5(RNAi)* did not enhance or suppress the *sep-1(RNAi)* centrosome duplication phenotype (Figures S2C–S2E). Given that centriole separation appears to proceed normally in mitotic divisions of separase-depleted embryos, we sought to investigate whether increased pericentriolar material and/or stronger microtubule-dependent pulling forces could compensate for loss of separase function. Recent work has implicated the kinesin-1 cargo adaptor KCA-1 in preventing accumulation of pericentriolar material during meiosis. RNAi-mediated depletion of KCA-1 results in premature centrosome maturation, with formation of centrosomal asters during meiosis [19]. Aside from premature centrosome maturation, RNAi-mediated depletion of KCA-1 did not result in any noticeable centrosome defects, and duplication proceeded normally (Figures 3A and 3B). However, depletion of KCA-1 alongside SEP-1 largely rescued the centriole separation and centrosome duplication phenotype of *sep-1(RNAi)* (Figures 3A and 3B and Table S1). To exclude the possibility of reduced efficacy of RNAi in double depletions, we depleted SEP-1 alongside HYLS-1, a protein with no apparent function in the early embryo [20]. As seen in Figure S2A, this did not affect severity of the separase phenotype. We next tested whether the increase in pericentriolar material might rescue *sep-1(RNAi)* centriole duplication defects by increasing microtubule-dependent pulling forces. At high concentrations, DMSO has been reported to promote microtubule stabilization [21]. Addition of DMSO to the culture medium resulted in strongly enhanced centrosomal microtubule nucleation and abrupt movements of centrosomes within the embryo. In *sep-1(RNAi)* embryos, DMSO largely restored centriole separation and centrosome duplication (Figures 3C and 3D and Table S1). Importantly, no pericentriolar material aggregates were observed under these conditions, with GFP foci unambiguously identified as centrosomes by their persistence and duplication from one cell cycle to the next, a conclusion supported by the use of a strain coexpressing the centriolar markers SAS-4 and SPD-2 (Figure S3A). Interestingly, a fraction of DMSO-treated embryos displayed overduplication, with multiple daughter centrioles forming successively on the same parent centriole. Thus, enhanced microtubule-dependent forces acting on centrosomes can substitute for separase function to drive centriole separation at the meiosis-mitosis transition.

In a striking parallel with sister chromatid cohesion, proteins of the cohesin complex have been localized to centrosomes and implicated in centriole cohesion in vertebrates, their depletion resulting in centrosome splitting and formation of multipolar spindles [3, 22]. However, others have noted that loss of spindle pole integrity occurs subsequent to loss of sister chromatid cohesion and is potentially indirect [23]. Further, a variety of conditions that induce prolonged mitotic checkpoint arrest result in loss of sister chromatid cohesion, with loss of spindle bipolarity as a secondary consequence [24, 25]. Perhaps the strongest evidence comes from artificially cleavable cohesin rendering centriole disengagement sensitive to expression of exogenous protease but insensitive to normal anaphase triggers [4]. However, again there are conflicting reports using similar approaches [7, 26]. In *C. elegans*, the core cohesin complex is composed of two SMC subunits, SMC-1 and SMC-3, and two non-SMC proteins, SCC-1 and SCC-3. During meiosis, the kleisin subunit SCC-1 is replaced by REC-8, COH-3, and COH-4, with partially overlapping functions in sister chromatid cohesion and pairing of homologous chromosomes [27]. Recent work has implicated these meiosis-specific cohesins and their loading factors in centriole cohesion during spermatogenesis [13].

**Figure 4:**
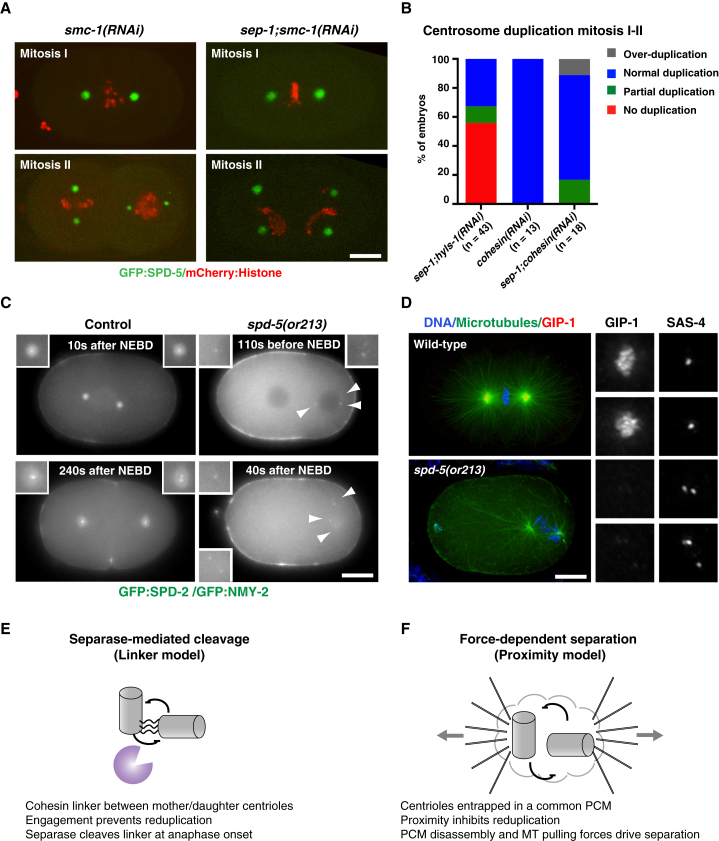
Cohesin and Pericentriolar Material Function in Centriole Cohesion at Different Stages in Embryonic Development (A) Stills from spinning-disk confocal time-lapse movies of *smc-1(RNAi)* and *smc-1;sep-1(RNAi)* embryos expressing GFP:SPD-5 and mCherry:Histone. (B) Quantitation of centrosome duplication phenotype of *cohesin(RNAi)* and *sep-1;cohesin(RNAi)* embryos. *sep-1;hyls-1(RNAi)* data are shown for comparison. Differences in duplication outcome between *sep-1;cohesin(RNAi)* and *sep-1;hyls-1(RNAi)* are statistically significant (Chi-square test, p < 0.05). (C) Stills from wide-field time-lapse sequences of control and *spd-5(or213)* mutant embryos expressing GFP:SPD-2 and GFP:myosin shifted to the restrictive temperature during pronuclear migration (S phase). Two independent examples are shown for *spd-5*. Arrowheads indicate centrioles. Note that centriole movement in *spd-5* mutants is not constrained after separation. (D) Immunofluorescence micrographs of wild-type and *spd-5(or213)* embryos stained for DNA, microtubules, pericentriolar material (GIP-1), and centrioles (SAS-4). Five centrioles are visible in the early prometaphase-stage *spd-5* embryo, indicating premature separation and overduplication. (E) In the original model for centriole duplication licensing, separase-mediated cleavage of a physical linker (potentially cohesin) between mother and daughter centrioles brings about centriole disengagement and permits reduplication. (F) In the alternative model, centrioles are held together by a common pericentriolar material until cortical pulling forces coupled with pericentriolar material disassembly drive centriole separation in anaphase. Reduplication is prevented by short-range inhibitory signals generated by the centrioles themselves. These mechanisms are not mutually exclusive: proximity due to linker-mediated engagement (rather than tethering per se) could inhibit reduplication and separase function could promote pericentriolar material disassembly in anaphase. In *C. elegans*, separase-mediated cohesin removal promotes centriole separation at the meiosis/mitosis transition, while cohesin- and separase-independent pericentriolar material entrapment mediates centriole cohesion at later stages. Scale bars represent 10μm; insets are magnified 2× (C) and 3.3× (D).

To circumvent the complications of multiple kleisins, we focused on the common subunits SMC-1 and SMC-3. Depletion of either protein by RNAi strongly perturbed chromosome dynamics, with impaired bivalent formation in meiosis and failure of chromosomes to align on the metaphase plate in mitosis (Figures S3D and S3E). Given that a cohesin-based centriole linker would presumably be established during centriole duplication in spermatogenesis, which is unaffected by RNAi [14], we did not expect to see premature disengagement of sperm centrioles after *smc-1* or *smc-3(RNAi)*, and indeed, sperm centriole separation was normal. However, no centrosomal phenotypes were evident in subsequent divisions (Figure 4A) or in a conditional *smc-3* mutant raised at the restrictive temperature (Figure S3F). These results argue against an essential function for cohesin in centriole cohesion in mitosis. Depletion of SMC-1 from *sep-1(RNAi)* embryos, however, resulted in near-complete rescue of centriole separation and centrosome duplication (Figures 4A and 4B and Table S1). Thus, separase-mediated cohesin removal does appear to be critical for centriole separation at the meiosis-mitosis transition. However, given that RNAi primarily affects maternally contributed protein, this cohesin appears to be of oocyte origin and not introduced with the sperm centriole pair at fertilization. Consistent with this idea, fertilization by *rec-8* mutant males did not rescue centriole separation defects in *sep-1(RNAi)* embryos (Figures S3B and S3C). Thus, separase is required to remove maternally loaded cohesin that is deposited after fertilization. While meiotic cohesin may help maintain centriole cohesion during spermatogenesis [13], our data suggest that this cohesin is exchanged in the zygote.

The above results indicate that separase and cohesin do not play a significant role in centriole cohesion beyond meiosis. Yet, during the centrosome duplication cycle in the *C. elegans* embryo as elsewhere, new daughter centrioles remain closely associated with their parent from their assembly in S phase until the end of mitosis. What mediates this close apposition? It is notable that this period of assembly and engagement coincides with the presence of substantial and increasing amounts of pericentriolar material surrounding the mother-daughter pair [14]. Pericentriolar material disassembly coincides with release of the newly assembled daughter centriole. We therefore sought to test whether entrapment in a common pericentriolar material could play a role in centriole cohesion in mitosis. Since the pericentriolar material is itself required for daughter centriole assembly [16], any subsequent role in centriole cohesion is difficult to address by RNAi. We found that we could bypass this requirement using a fast-acting temperature-sensitive mutation in *spd-5* [28]. To test pericentriolar material function in centriole cohesion, we grew embryos expressing GFP:SPD-2 and the cortical marker GFP:myosin at the permissive temperature of 16°C and then shifted them to 25°C at different times in the first cell cycle. Whereas in control embryos centriole separation invariably occurred after anaphase onset and coincident with cytokinesis (8 ± 18 s before cleavage furrow ingression, 125 ± 26 s after anaphase onset, or 238 ± 28 s after NEBD), in more than half of mutant embryos at the restrictive temperature one or both mother-daughter centriole pairs separated prematurely (16/26 embryos, 89 ± 87 s before NEBD; Figure 4C). This is likely to be an underestimate given the relatively low spatial resolution of our live-imaging conditions. Immunofluorescence microscopy confirmed this result and also revealed signs of overduplication, indicating a failure of proper duplication control (Figure 4D).

Our results lead us to a number of conclusions regarding centriole cohesion and duplication licensing in *C. elegans* (see also Figures 4E and 4F). First, there does not appear to be a single dedicated linker connecting mother and daughter centrioles. Cohesin contributes to maintenance of sperm centriole cohesion after fertilization until completion of female meiosis and its separase-mediated removal is required to fully separate centrioles. Pericentriolar material maintains mother-daughter centriole cohesion during subsequent mitoses, and its removal by microtubule-based pulling forces likely contributes to centriole separation. Differences in developmental context could in part explain the discrepancies between results obtained in different experimental models, with studies supporting a role for cohesin in centriole cohesion conducted primarily in meiotic or early embryonic systems [4, 13], while studies reporting cohesin to be dispensable were conducted in mitotic embryos or somatic cells [7, 26]. This correlation is not absolute, and it is possible that cohesin could play a minor role at all stages. A role for the pericentriolar material in centriole cohesion is not unanticipated, and pericentriolar material disassembly upon mitotic exit (potentially mediated by separase in some organisms [29, 30]) presents an attractive mechanism to coordinate centriole separation with the cell cycle. In principle, an intercentriolar linker could be embedded within the pericentriolar material. However, laser ablations in vertebrate cells have shown that movement of two daughters formed on the same mother remains coordinated even after ablation of the parent [31], a result incompatible with a linker structure tethering mother and daughter centrioles but consistent with matrix entrapment. Additional mechanisms to control centriole engagement likely exist: in vertebrates, Plk1 phosphorylation has been shown to play an important role in centriole maturation, a necessary step for subsequent disengagement [32]. In our *spd-5* experiments, premature centriole separation was only observed close to mitosis, hinting at a similar licensing step. We were unable to examine this further, given the essential functions for PLK-1 in gonad development and embryo production [33]. Second, centrioles do not need to be engaged to inhibit each other’s duplication potential, but rather appear to be able to do so below a certain distance from each other. These results lead us to favor a proximity inhibition model whereby centrioles impair each other’s duplication potential unless sufficiently separated, possibly by generating a short-range diffusible inhibitory signal. Such a model was recently proposed by Wang et al. [32], although we would suggest that both mature (duplication competent) and immature centrioles would have to be able to generate such a signal to explain the mutual interference observed in our experiments. Relief from proximity inhibition could also explain the formation of multiple daughter centrioles distal from each other on the same overelongated mother centriole in vertebrate cells [34]. Importantly, this model does not depend on the existence of a physical structure connecting centrioles that requires specific disassembly or cleavage.

It is clear from our data that separase does not play an essential role in centriole separation in *C. elegans* mitosis. Similarly, loss of separase does not affect centrosome duplication in *Drosophila* embryos [5] and merely delays centriole separation in vertebrate somatic cells [7]. By contrast, separase function does appear to be critical at the meiosis-mitosis transition in *C. elegans* (this study) and in the in vitro experiments in *Xenopus* extracts [2], both cases in which microtubule forces are weak or absent. As has been suggested for separase function in cytokinesis [35] an indirect mechanism via Cdk1 inhibition could conceivably be at work, and indeed separase RNAi embryos suffer extensive delays in exit from meiosis [12] (Figures S4A and S4B). However, codepletion of cohesin rescued centriole separation without restoring normal cell-cycle timing, arguing for a cell-cycle-independent effect of separase (Figure S4B). Whether the apparent target of separase activity, cohesin, localizes to centrosomes remains unclear since we were unable to reliably detect cohesin in the early embryo (data not shown). Since separase is enriched on sperm chromatin at the time of centrosome separation (Figure S1), we excluded indirect effects on sperm centrioles via the chromatin using anucleated sperm (*emb-27* [36]), which did not affect the centriole separation defect of *sep-1(RNAi)* embryos (Figures S4C and S4D), despite the presence of extra centrioles delivered by mutant sperm [11]. These results provide strong evidence for a direct effect of separase on centriole cohesion at the meiosis/mitosis transition.

Whether cohesin acts as a true intercentriolar linker or as an entrapment matrix similar to the pericentriolar material remains to be determined. However, the conceptually appealing view of separase cleaving a single cohesin-based linker to coordinate centrosome and chromosome cycles will need to be reconsidered. Instead, our work points to multiple mechanisms contributing to centriole engagement and separation, with the pericentriolar material playing a central role in mitosis.

## Supplemental Information

Document S1. Supplemental Experimental Procedures, Figures S1–S4, and Table S1
