# Supplementary material for: Multiple Mechanisms Contribute to Centriole Separation in C. elegans
Source: Curr Biol. 2013 Jul 22;23(14):1380–7. doi: 10.1016/j.cub.2013.06.043 (PMC3722485; doi:10.1016/j.cub.2013.06.043)
Supplement: Document S1. Supplemental Experimental Procedures, Figures S1–S4, and Table S1 [file mmc1.pdf]

Current Biology, Volume *23*

Supplemental Information

Multiple Mechanisms Contribute

to Centriole Separation in *C. elegans*

Gabriela Cabral, Sabina Sanegre Sans, Carrie R. Cowan, and Alexander Dammermann

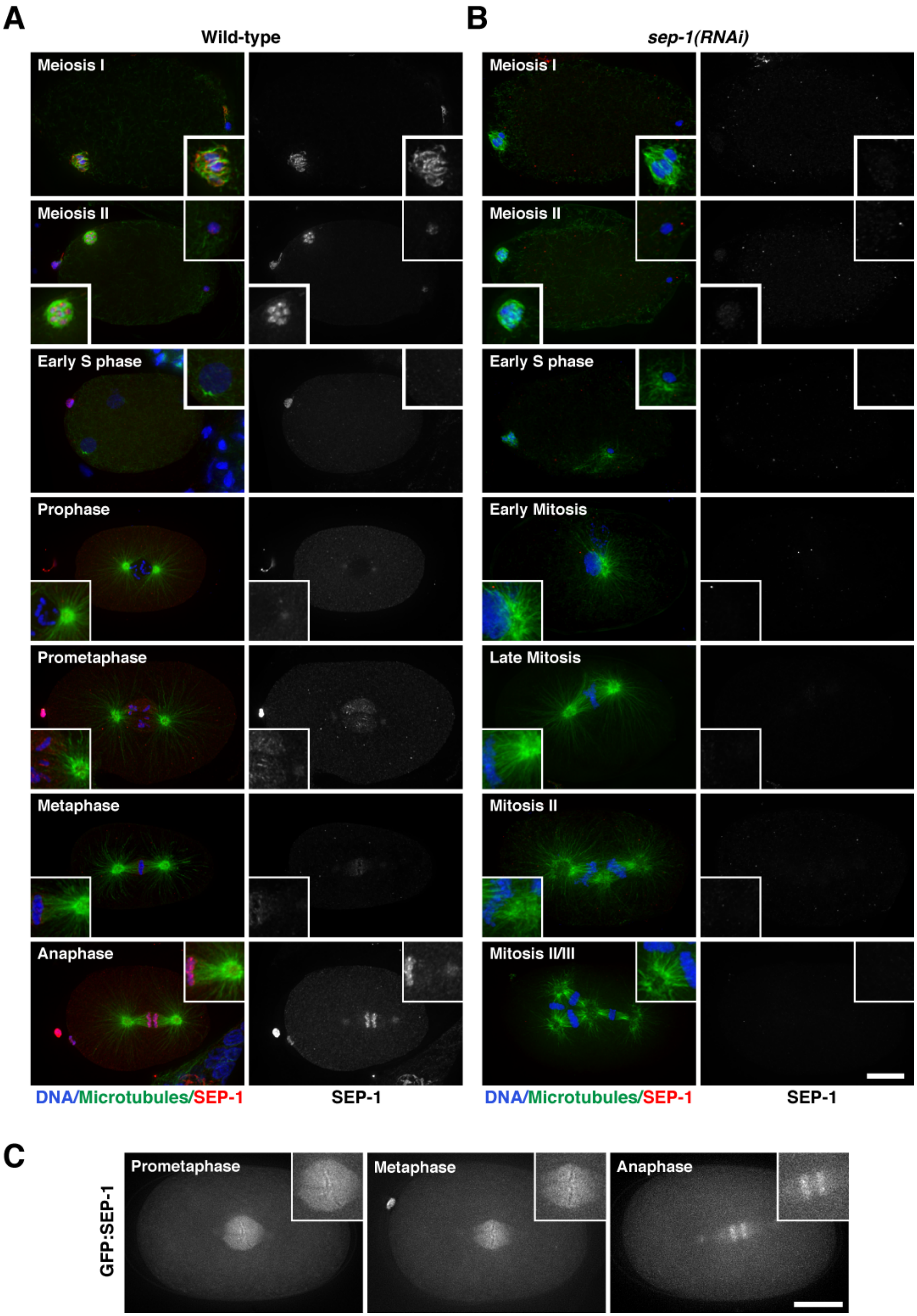

Figure S1. Localization of separase in *C. elegans* embryos, Related to the Results and Discussion

Immunofluorescence micrographs of wild-type (**A**) and *sep-1(RNAi)* (**B**) embryos stained for DNA, microtubules and SEP-1. Insets show meiotic spindle, sperm pronucleus and later mitotic

spindle. Separase localizes to meiotic and mitotic chromosomes, the spindle as well as mitotic centrosomes. No change in separase signal is seen at centrosomes from prophase to anaphase. At chromosomes, separase appears to spread from the holocentric centromere/kinetochore in prometaphase and metaphase to throughout the chromatin in anaphase. No residual signal was seen in *sep-1(RNAi)* embryos, indicating that staining is specific and depletion efficient. All embryos were exposed and processed identically for separase, except for control meiosis embryos, where exposure conditions were reduced 3x to avoid saturating signal. (**C**) Stills from widefield timelapse sequence of control embryo expressing GFP:SEP-1, showing redistribution of separase at anaphase onset. Scalebars are 10μm. Insets are magnified 2x (**A**, **B**), 1.5x (**C**).

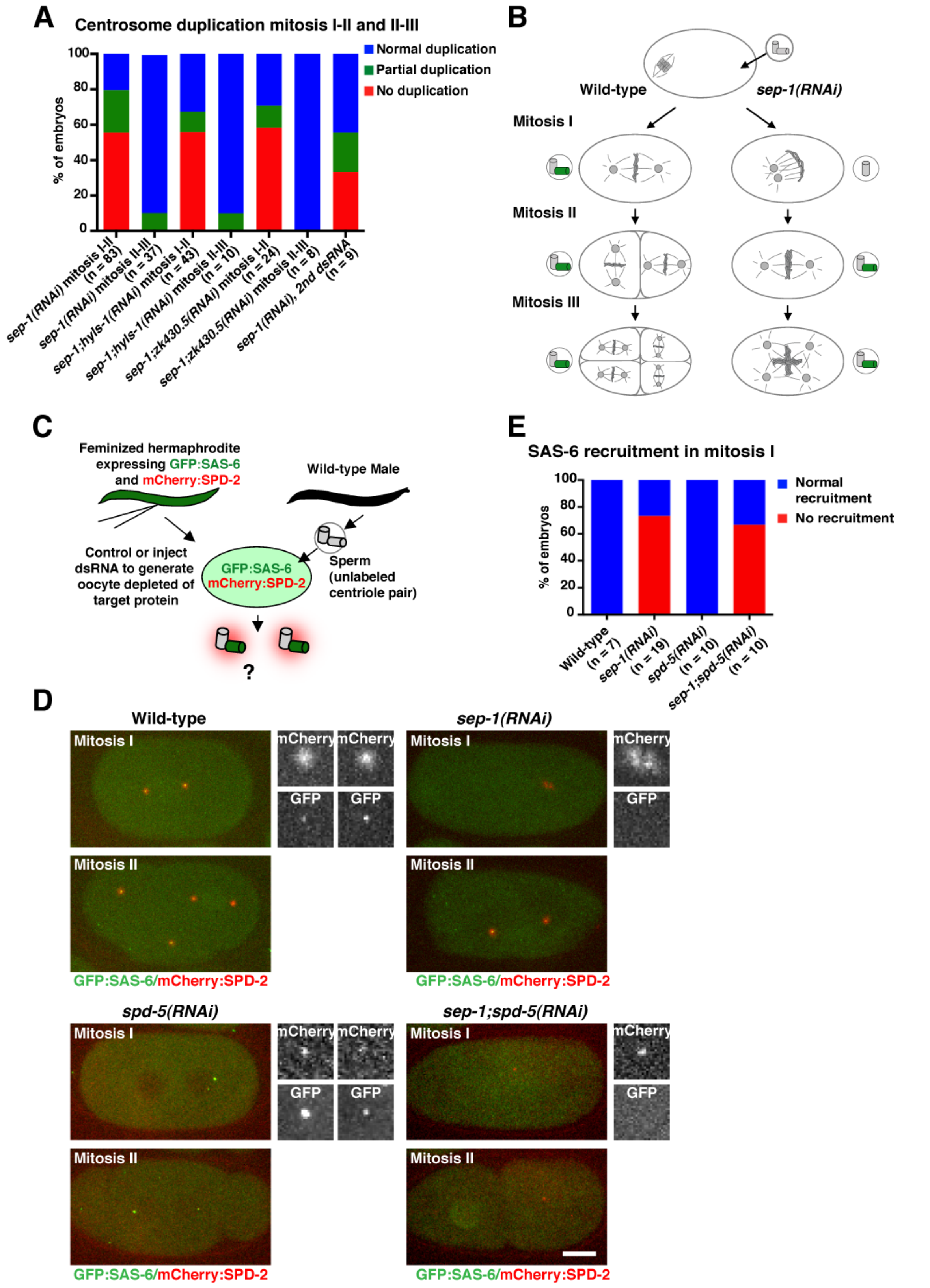

Figure S2. Further analysis of the separase RNAi phenotype, Related to the Results and Discussion

(**A**) Quantitation of centrosome duplication phenotype in *sep-1* single depletions, double depletions with dsRNAs targeting *hyls-1* (control), *zk430.5* (a non-essential gene related to sep-1)

and single depletions using a second, non-overlapping dsRNA targeting *sep-1*, based on timelapse sequences as in Figure 1A. Centrosome duplication outcome is not significantly different between any of these conditions (Chi-square test, $p>0.05$). (**B**) Schematic representation of centrosome and centriole dynamics in wild-type and *sep-1(RNAi)* embryos. In wild-type embryos, a pair of centrioles is introduced into the oocyte with the sperm at fertilization. Sperm-derived centrioles separate and a new daughter centriole assembles alongside each parent such that by metaphase each centrosome contains two centrioles, one sperm-derived and one newly assembled in the embryo cytoplasm (in schematics parental centrioles are shown in grey, daughters in green). At the end of mitosis these centrioles separate and a new round of assembly occurs. In *sep-1(RNAi)* embryos, since spermatogenesis is complete prior to application of dsRNA, a normal pair of centrioles still enters the oocyte at fertilization. However, with the oocyte cytoplasm depleted of separase, centrioles do not separate sufficiently in the first mitosis. Consequently, monopolar spindles form and centriole duplication is blocked. At the end of mitosis I as well as in subsequent mitotic cycles, centrioles move apart and inhibition of centriole duplication is relieved. Since cytokinesis frequently fails, successive mitotic cycles occur in the same one-cell embryo, with two centrosomes in mitosis II (now comprised of a pair of centrioles) and four in mitosis III. (**C**) - (**E**) The pericentriolar material does not contribute to sperm centriole cohesion at the meiosis/mitosis transition. (**C**) Mating-based assay for SAS-6 recruitment. Similar to centriole duplication assay outlined in Figure 2A, except using SAS-6 in place of SAS-4. SAS-6 recruitment occurs at an earlier step in the centriole assembly pathway unaffected by depletion of γ-tubulin and therefore likely also by depletion of SPD-5[1]. Note that recruitment of SAS-6 (unlike that of SAS-4) does not indicate completion of centriole assembly. (**D**) Results of experiments performed as outlined in (**C**) on control, *sep-1(RNAi)*, *spd-5(RNAi)*, and *sep-1;spd-5(RNAi)* embryos, using spinning disk confocal microscopy. As expected, depletion of SPD-5 did not affect SAS-6 recruitment and initiation of centriole assembly. However, *sep-1(RNAi)* did perturb initiation of centriole assembly, to an extent not significantly altered by co-depletion of SPD-5 (Chi-square test, $p>0.05$). (**E**) Quantitation of centriole duplication assay shown in (**D**). Scalebars in (**D**) are 10μm, insets are magnified 4x.

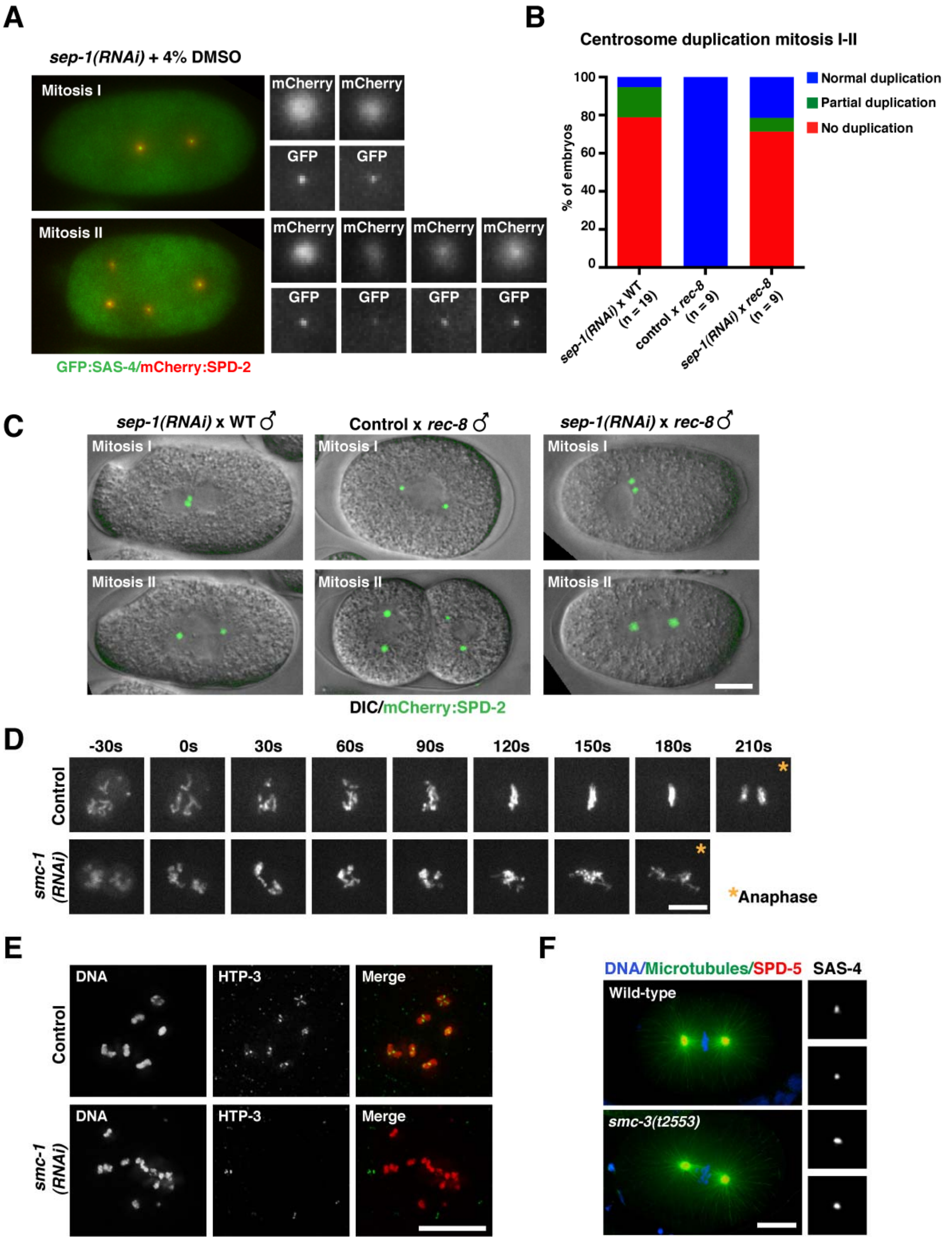

Figure S3. DMSO rescue and further analysis of the cohesin phenotype, Related to the Results and Discussion

(**A**) DMSO treatment restores centriole separation and duplication in *sep-1(RNAi)* embryos. Stills from widefield timelapse sequences of embryos co-expressing GFP:SAS-4 and mCherry:SPD-2. Note the absence of any appreciable non-centrosomal pericentriolar material accumulation following DMSO treatment (n=15 embryos). (**B**) Fertilization of *sep-1(RNAi)* embryos with cohesin mutant sperm does not rescue centriole duplication. Quantitation performed on timelapse sequences acquired as outlined in Figure 2A, except using *rec-8(ok978)* males. Feminized

hermaphrodites were used to eliminate the possibility of self-fertilization. Centriole duplication outcome is not significantly different compared to *sep-1(RNAi)* alone (Chi-square test, $p>0.05$). (**C**) Stills from spinning disk confocal timelapse sequences of embryos resulting from the indicated crosses, showing an overlay of DIC and SPD-2 images. (**D**) Cohesin depletion results in chromosome segregation defects in mitosis. Stills of timelapse spinning disk confocal movies of control and *smc-1(RNAi)* embryos expressing mCherry:Histone, aligned relative to nuclear envelope breakdown (t=0 seconds). Chromosomes fail to form a metaphase plate and display severe defects in anaphase segregation (n=12 embryos). (**E**) Cohesion depletion results in failure of sister chromatin cohesion during meiosis. Immunofluorescence micrographs of nuclei from diakinesis-stage wild-type and *smc-1(RNAi)* oocytes stained for DNA and HTP-3. (**F**) Cohesin mutants do not display premature centriole separation in mitosis. Immunofluorescence micrographs of wild-type and *smc-3(t2553)* mutant embryos stained for DNA, microtubules and SPD-5, as well as SAS-4 (insets). Scalebars are 10µm. Insets in (**A**) and (**F**) are magnified 4x.

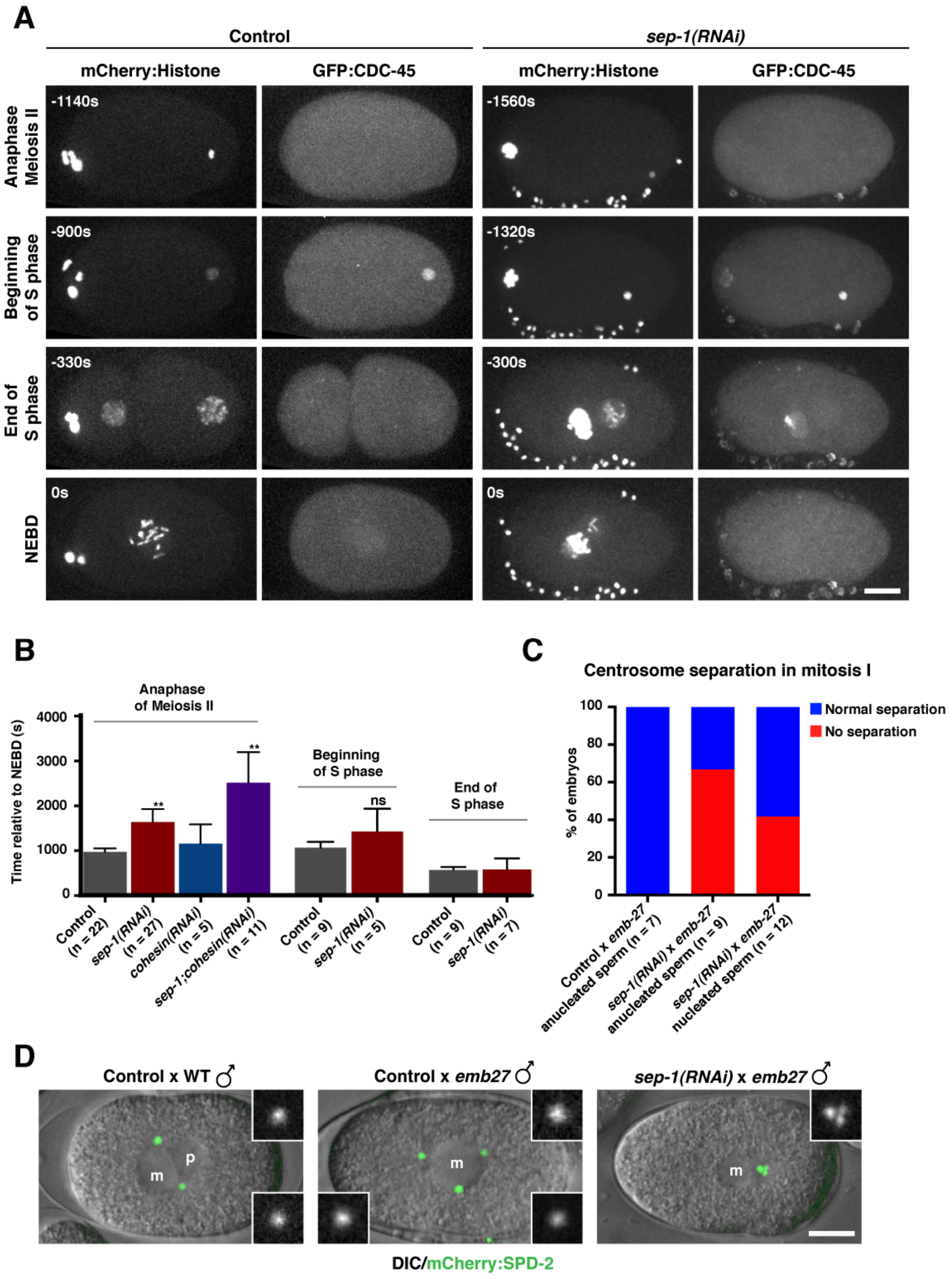

Figure S4. Separase function in centriole separation may be direct, Related to the Results and Discussion

(**A**) Stills from spinning disk confocal timelapse movies of control and *sep-1(RNAi)* embryos expressing the S-phase marker GFP:CDC-45 and mCherry:Histone, depicting key events relevant to centriole assembly. (**B**) Timing analysis performed on time-lapse movies of control, *sep-1(RNAi), smc-1(RNAi)* and *smc-1;sep-1(RNAi)* embryos expressing GFP:CDC-45 and/or mCherry:Histone. In *sep-1(RNAi)* embryos, anaphase onset was scored as onset of attempted

chromosome segregation. Beginning and end of S-phase were assessed by recruitment and loss of GFP:CDC-45, measured as described in [2]. Error bars represent the 95% confidence interval. Asterisks indicate statistically significant differences from control (t-test, $p<0.05$; ns not significant). Note that *sep-1(RNAi)* embryos experience significant delays in entry into S-phase after exit from meiosis. However, duration of S-phase and subsequent entry into mitosis (nuclear envelope breakdown, NEBD) are largely normal. SMC-1 co-depletion rescues the *sep-1(RNAi)* centrosome duplication phenotype (Figure 4A, B) without restoring normal cell cycle timing. (**C**, **D**) The presence or absence of sperm chromatin does not affect sperm centriole separation in control and *sep-1(RNAi)* embryos. Mating of feminized hermaphrodites with *emb-27(g48)* mutant males raised at the restrictive temperature of 25.5C frequently results in fertilization with anucleated sperm (identifiable by the absence of a sperm pronucleus by DIC). Centrosome separation was assessed in a strain expressing mCherry:SPD-2 and GFP:SAS-4 in control and *sep-1(RNAi)* embryos. (**C**) Quantitation of centriole separation phenotype. (**D**) Stills from spinning disk confocal timelapse sequences of embryos resulting from the indicated crosses, showing an overlay of DIC and SPD-2 images. Maternal and paternal pronuclei are labeled ‘m’ and ‘p’. Insets show SPD-2 only. Note that *emb-27* mutants frequently introduce supernumerary centrioles. Centriole separation was normal in control embryos, but frequently failed in *sep-1(RNAi)* embryos, irrespective of the presence or absence of sperm chromatin. Scalebars are 10μm. Insets in (**D**) are magnified 1.5x.

Table S1. Centriole separation statistics for S phase (-650 to -350s), Related to Figure 1

	Average separation (+/- 95% confidence interval)	Range (min/max)	Number of embryos examined	Statistically significant? (t-test <0.05)
Wild-type	3.2 +/- 1.0µm	0.3 – 6.8µm	15	n.a.
sep-1(RNAi) (all outcomes)	1.2 +/- 0.3µm	0 – 7.8µm	51	Yes cf. wild-type
sep-1(RNAi) (no duplication)	0.6 +/- 0.2µm	0 – 1.5µm	20	Yes cf. wild-type
sep-1(RNAi) (partial duplication)	1.2 +/- 0.2µm	0.6 – 2.1µm	14	Yes cf. no duplication
sep-1(RNAi) (normal duplication)	1.9 +/- 1.0µm	0.5 – 7.8µm	17	Yes cf. no duplication
sep-1;kca-1(RNAi)	2.6 +/- 1.6µm	1.1 – 9.1µm	11	Yes cf. *sep-1(RNAi)*
sep-1(RNAi)+DMSO	12.3 +/- 1.6µm	9.4 – 14.3µm	8	Yes cf. *sep-1(RNAi)*
sep-1;smc-1(RNAi)	7.2 +/- 4.3µm	1.4 – 14.7µm	9	Yes cf. *sep-1(RNAi)*

SUPPLEMENTAL EXPERIMENTAL PROCEDURES

C. elegans strains and culture conditions

Strains expressing GFP:CDC-45[2], SAS-6:GFP[3], SEP-1:GFP[4] and mCherry:H2B[5] have been described previously. The strain expressing mCherry:SPD-2 was generated by cloning the corresponding genomic locus into pAA65[5] and ballistic bombardment[6]. Strains expressing GFP:SAS-4 and GFP:SPD-5 were generated by cloning the corresponding genomic loci including 5' and 3' regulatory sequences and N-terminal GFP into the chromosome II targeting vector pCFJ151 and Mos1-mediated transposition[7]. Dual color and recruitment strains carrying the temperature-sensitive female-sterile mutation *fem-1(hc17)* were constructed by mating. The genotypes of all strains used are listed in table below. Strains were maintained at 16C (DAM373, DAM466, EU856, GG48, UE21 UE31) or 23C (others). Recruitment analysis was performed using N2 males as described[8], with mCherry:SPD-2 in strains DAM373 and DAM466 serving to label sperm centrioles from immediately after fertilization. Cohesin mutants were maintained as balanced heterozygotes. Analyses were performed on homozygous offspring negative for the GFP-marked balancer. *rec-8(ok978)* is a deletion reported to be a strong loss of function or null mutant[9]. The temperature-sensitive mutants *smc-3(t2553)*[10] and *emb-27(g48)*[11] were shifted to the restrictive temperature of 25C at L1, while *spd-5(or213)* mutants[12] were kept at 16C until immediately prior to the experiment.

RNA-mediated interference

RNAi experiments were performed by injection[1] or soaking[13] using the dsRNAs listed in table below. For double depletions, RNAs were mixed at equal concentrations of ≥1 mg/ml for each RNA, with HYLS-1 used as a control in corresponding single depletions. Standard RNAi conditions of 48h at 16C (72h for SMC-1/SMC-3) were used to ensure full depletion. For RNAi experiments involving anucleated sperm, injected worms were mated to *emb-27(g48)* mutant males for 24h at 25C to avoid reversion of the *emb-27* phenotype, before removing males and returning hermaphrodites to 16C for the remaining 24h.

Immunofluorescence and fixed imaging

Immunofluorescence experiments were performed using directly-labelled affinity-purified antibodies to α-tubulin (DM1α-FITC, Sigma), GIP-1[14], SAS-4[8], SEP-1 (C-terminus, amino acids 1064-1262; this study) and SPD-5[8], as well as unlabeled antibodies to HTP-3[15] as previously described[16]. 3D widefield datasets were acquired using a 100X 1.4NA Super Plan Apochromat lens on a DeltaVision microscope equipped with a 7-Color SSI module and CoolSNAP-HQ2 cooled CCD camera, computationally deconvolved and projected using SoftWorx (Applied Precision), before being imported into Adobe Photoshop for final processing.

Live imaging

Embryos were filmed without compression[17] under attenuated fluorescence illumination conditions known not to perturb cell cycle progression. Widefield microscopy was performed using a 60x 1.42 NA Plan Apochromat lens on the DeltaVision microscope set-up described above. Spinning disk confocal microscopy was performed on a Yokogawa CSU X1-A1 spinning disk confocal mounted on a Zeiss Axio Observer Z1 inverted microscope equipped with a 63x 1.4NA Plan Apochromat lens, 100mW 488nm and 561nm solid-state lasers and CoolSNAP-HQ2 cooled CCD camera and controlled by VisiView software (Visitron Systems). For embryos co-expressing GFP:SPD-5 and mCherry:Histone, 6x2µm GFP/mCherry z-series as well as single plane DIC images were acquired every 30s from meiosis until the end of the second or third mitotic division. For embryos co-expressing GFP:CDC-45 and mCherry:Histone, 6x2µm GFP/mCherry z-series as well as single plane DIC images were acquired every 30s from meiosis until NEBD, with 2x2 camera binning used to enhance signal detection. For SAS-4 and SAS-6 recruitment analysis, 11x1µm GFP/mCherry z-series as well as single plane DIC images were

acquired every 60s from early prophase until the end of the second mitotic division, with 2x2 binning to enhance signal detection. Image stacks were imported into MetaMorph for post-acquisition processing. Panels are single-plane or maximum intensity projections of multiple planes depending on centrosome positioning within the embryo. Images are auto-scaled for best presentation unless otherwise noted.

Temperature shift experiments

spd-5(or213) temperature shift experiments were performed on a Zeiss Axioplan2 widefield microscope with a temperature-controlled 40x 1.3 NA Plan-Neofluar oil lens. Lens temperature was achieved by a custom 'cooling' collar (Bioptechs) that was heated by circulating antifreeze; antifreeze temperature was controlled by a circulating water bath. Images were acquired with a CoolSnap HQ (Photometrics) camera, using 488nm LED illumination (CoolLED, Andover). Image acquisition was controlled by VisiView (Visitron Systems). Worms were maintained at 16C prior to sample preparation. One-cell embryos were located at 10x magnification and ambient temperature (~20C) then shifted within one minute to the 40x lens at 25C. The temperature of the sample equilibrated to the lens temperature in less than two minutes, as judged by a temperature probe mounted as for embryos. 25x1µm GFP z-series and single plane DIC images were acquired at irregular intervals from immediately after the shift to 25C until the end of mitosis I.

Centriole separation measurements and statistical analyses

Centriole separation was measured on projected images in MetaMorph, disregarding any separation in the z-dimension. Each embryo was scored once per 100s interval. For the analysis in Table S1, distances were further averaged across the entire S phase interval such that each embryo is represented once in the overall average. Statistical comparisons were performed in Excel (Student's t-test, two-tailed), without disregarding any datapoints. Comparisons of centriole duplication outcome were performed in GraphPad Prism (Chi-square test) with data grouped into three categories (no duplication, partial duplication, normal duplication). Results were reported as significant if p value was <0.05.

C. elegans strains used in this study

Strain #	Genotype
DAM349	vieSi15 [pAD395; Pspd-5/GFP-spd-5reencoded; cb unc-119(+)] II; ltIs37 [pAA64; Ppie-1/mCherry::his-58; unc-119 (+)] IV
DAM373	vieSi18 [pAD154; Psas-4/GFP:sas-4reencoded; cb unc-119(+)] II; sas-4(tm3951) III; fem-1(hc17); ltIs69 [pAA191; Ppie-1/mCherry-spd-2; unc-119 (+)] IV
DAM466	ltSi40 [pOD1227; Psas-6/sas-6reencoded:GFP; cb unc-119(+)] II; fem-1(hc17); ltIs69 [pAA191; pie-1/mCherry-spd-2; unc-119 (+)] IV
EU856	spd-5(or213)I
GG48	emb-27(g48)II
N2	C. elegans wild-type (ancestral)
TG1754	unc-119(ed3) III; gtIs65 [Ppie-1/GFP-cdc-45; unc-119(+)]; ltIs37 [Ppie-1-mCherry-his-58; unc-119(+)]
UE21	unc-119(ed3) III; ddIs10 [GFP::SPD-2 genomic; unc-119(+)]; zuIs45 [nmy-2::NMY-2::GFP + unc-119(+)]
UE32	spd-5(or213)I; unc-119(ed3) III; ddIs10 [GFP::SPD-2 genomic; unc-119(+)]; zuIs45 [nmy-2::NMY-2::GFP + unc-119(+)]
UV97	smc-3(t2553)/hT2 [bli-4(e937)let-?(q782)qIs48](I;III)
VC666	rec-8(ok978) IV/nt1 (qls51) (IV;V)
WH416	unc-119(ed3) III; ojIs58 [pie-1p::sep-1::GFP + unc-119(+)]

dsRNAs used in this study

Gene	Name	mg/ml	Oligo #1	Oligo #2	Template
C05C8.9	*hyls-1*	2.1	AATTAACCCTCACTAAAGGT GGCAAATTTTACCACTGAAA	TAATACGACTCACTATAGGTG ATATCTTGTGACCGGATCA	N2 genomic DNA
C10H11.10	*kca-1*	2.5	AATTAACCCTCACTAAAGGA TGCATTCGTCGAAACTTCC	TAATACGACTCACTATAGGGG CGTCAGACTGAGAAGGAC	N2 cDNA
Y45F10D.9	*sas-6*	4.7	AATTAACCCTCACTAAAGGC CGCTCCGATGATTTTGAAT	TAATACGACTCACTATAGGCC AAGAACAGGCTTGAATGA	N2 genomic DNA
Y47G6A.12	*sep-1*	2.3	AATTAACCCTCACTAAAGGA ATCGCTCCCAACAGAATTG	TAATACGACTCACTATAGGAG TTTCCCATGGAATGCAAC	N2 cDNA
Y47G6A.12	*sep-1* (2nd RNA)	1.8	AATTAACCCTCACTAAAGGTT TCGGGATCTTGATGGAGT	TAATACGACTCACTATAGGTC GACGTACAAAACGCCTAC	N2 genomic DNA
ZK430.5	*N/A*	2.0	AATTAACCCTCACTAAAGGA CTGGCTAAAAGCCGAGTGA	TAATACGACTCACTATAGGTT GAAATACCGGACGACAGAT	N2 genomic DNA
F28B3.7	*smc-1*	2.4	AATTAACCCTCACTAAAGGC AGCAGAACCTCCGGACATA	TAATACGACTCACTATAGGAA GGCAGAGAACAACTCGACTC	N2 genomic DNA
Y47D3A.26	*smc-3*	1.5	AATTAACCCTCACTAAAGGA TGAAGATCAAAGAAGTG	TAATACGACTCACTATAGGGT TTCCTTCAGGATTTTC	N2 genomic DNA
F56A3.4	*spd-5*	2.3	AATTAACCCTCACTAAAGGT GTCGCAACCAGTTCTGAAT	TAATACGACTCACTATAGGAT GGAGGCAAATTGTTGCTG	N2 genomic DNA

SUPPLEMENTAL REFERENCES

1. Dammermann, A., Maddox, P.S., Desai, A., and Oegema, K. (2008). SAS-4 is recruited to a dynamic structure in newly forming centrioles that is stabilized by the gamma-tubulin-mediated addition of centriolar microtubules. J Cell Biol *180*, 771-785.
2. Sonneville, R., Querenet, M., Craig, A., Gartner, A., and Blow, J.J. (2012). The dynamics of replication licensing in live Caenorhabditis elegans embryos. J Cell Biol *196*, 233-246.
3. Qiao, R., Cabral, G., Lettman, M.M., Dammermann, A., and Dong, G. (2012). SAS-6 coiled-coil structure and interaction with SAS-5 suggest a regulatory mechanism in C. elegans centriole assembly. EMBO J *31*, 4334-4347.
4. Bembenek, J.N., Richie, C.T., Squirrell, J.M., Campbell, J.M., Eliceiri, K.W., Poteryaev, D., Spang, A., Golden, A., and White, J.G. (2007). Cortical granule exocytosis in C. elegans is regulated by cell cycle components including separase. Development *134*, 3837-3848.
5. McNally, K., Audhya, A., Oegema, K., and McNally, F.J. (2006). Katanin controls mitotic and meiotic spindle length. J Cell Biol *175*, 881-891.
6. Praitis, V., Casey, E., Collar, D., and Austin, J. (2001). Creation of low-copy integrated transgenic lines in Caenorhabditis elegans. Genetics *157*, 1217-1226.
7. Frokjaer-Jensen, C., Davis, M.W., Hopkins, C.E., Newman, B.J., Thummel, J.M., Olesen, S.P., Grunnet, M., and Jorgensen, E.M. (2008). Single-copy insertion of transgenes in Caenorhabditis elegans. Nat Genet *40*, 1375-1383.
8. Dammermann, A., Muller-Reichert, T., Pelletier, L., Habermann, B., Desai, A., and Oegema, K. (2004). Centriole assembly requires both centriolar and pericentriolar material proteins. Dev Cell *7*, 815-829.
9. Severson, A.F., Ling, L., van Zuylen, V., and Meyer, B.J. (2009). The axial element protein HTP-3 promotes cohesin loading and meiotic axis assembly in C. elegans to implement the meiotic program of chromosome segregation. Genes Dev *23*, 1763-1778.
10. Baudrimont, A., Penkner, A., Woglar, A., Mamnun, Y.M., Hulek, M., Struck, C., Schnabel, R., Loidl, J., and Jantsch, V. (2011). A new thermosensitive smc-3 allele reveals involvement of cohesin in homologous recombination in C. elegans. PLoS One *6*, e24799.
11. Sadler, P.L., and Shakes, D.C. (2000). Anucleate Caenorhabditis elegans sperm can crawl, fertilize oocytes and direct anterior-posterior polarization of the 1-cell embryo. Development *127*, 355-366.
12. Hamill, D.R., Severson, A.F., Carter, J.C., and Bowerman, B. (2002). Centrosome maturation and mitotic spindle assembly in C. elegans require SPD-5, a protein with multiple coiled-coil domains. Dev Cell *3*, 673-684.
13. Green, R.A., Kao, H.L., Audhya, A., Arur, S., Mayers, J.R., Fridolfsson, H.N., Schulman, M., Schloissnig, S., Niessen, S., Laband, K., et al. (2011). A high-resolution C. elegans essential gene network based on phenotypic profiling of a complex tissue. Cell *145*, 470-482.
14. Hannak, E., Oegema, K., Kirkham, M., Gonczy, P., Habermann, B., and Hyman, A.A. (2002). The kinetically dominant assembly pathway for centrosomal asters in Caenorhabditis elegans is gamma-tubulin dependent. J Cell Biol *157*, 591-602.
15. Goodyer, W., Kaitna, S., Couteau, F., Ward, J.D., Boulton, S.J., and Zetka, M. (2008). HTP-3 links DSB formation with homolog pairing and crossing over during C. elegans meiosis. Dev Cell *14*, 263-274.
16. Oegema, K., Desai, A., Rybina, S., Kirkham, M., and Hyman, A.A. (2001). Functional analysis of kinetochore assembly in Caenorhabditis elegans. J Cell Biol *153*, 1209-1226.
17. Monen, J., Maddox, P.S., Hyndman, F., Oegema, K., and Desai, A. (2005). Differential role of CENP-A in the segregation of holocentric C. elegans chromosomes during meiosis and mitosis. Nat Cell Biol *7*, 1248-1255.
